# C-Reactive Protein-to-Albumin Ratio as a Predictor of Short-Term Functional Outcomes After Spontaneous Intracerebral Hemorrhage: A Prospective Cohort Study

**DOI:** 10.7759/cureus.113766

**Published:** 2026-08-01

**Authors:** S M Arafath Amin, Pijush Paul, Md Mizanur Rahman, Md Amirul Islam, Sharmin Akhter, Md Ziaul Hoque Bablu, Mohiuddin Ahmed, Tasneem Jerin, Kazi Giasuddin Ahmad, Md. Shamsuzzaman

**Affiliations:** 1 Department of Neurology, National Institute of Neurosciences & Hospital, Dhaka, BGD; 2 Department of Interventional Neurology, National Institute of Neurosciences & Hospital, Dhaka, BGD; 3 Department of Neurology, Dhaka Medical College and Hospital, Dhaka, BGD; 4 Department of Medicine, 250 Bed General Hospital, Munshiganj, Munshiganj, BGD; 5 Department of Neurology, Dhaka Medical College, Dhaka, BGD; 6 Department of Palliative Medicine, Palliative Care Society of Bangladesh, Dhaka, BGD; 7 Department of Neurology, National Institute of Neurosciences and Hospital, Dhaka, BGD

**Keywords:** c-reactive protein-to-albumin ratio, functional outcome, inflammation, prognostic biomarker, spontaneous intracerebral hemorrhage

## Abstract

Background

The C-reactive protein-to-albumin ratio (CAR) is an emerging biomarker reflecting systemic inflammation and nutritional status. However, evidence regarding its prognostic value for short-term functional outcomes after spontaneous intracerebral hemorrhage (ICH), particularly in resource-limited settings, remains limited. This study aimed to investigate the associations between the early CAR and 30-day functional outcomes in patients with spontaneous ICH.

Methods

This prospective cohort study enrolled 250 adults with first-ever CT-confirmed spontaneous ICH and admission National Institutes of Health Stroke Scale (NIHSS) scores of 5-15 at two tertiary hospitals in Dhaka, Bangladesh, between February 2021 and January 2023. Of these, 220 patients with available 30-day outcome data were included in the complete-case analysis. Clinical, laboratory, and radiological data were collected, and the CAR was calculated from samples obtained during the initial laboratory evaluation after hospital presentation and within 72 hours of stroke onset. The primary outcome was 30-day functional status assessed using the modified Rankin scale (mRS), categorized as favorable (0-3) or unfavorable (4-6). Multivariable logistic regression identified independent predictors of unfavorable outcomes, and receiver operating characteristic analysis evaluated the discriminatory performance of CAR.

Results

Among the 220 patients, 130 (59.1%) experienced an unfavorable outcome. The CAR was moderately correlated with the mRS score (rs = 0.513, p < 0.001). According to the multivariate analysis, a higher CAR independently predicted unfavorable outcomes (adjusted OR 4.51; 95% CI 1.63-12.50; p = 0.004). Additional predictors included increasing age, hemiparesis, aspiration pneumonia, hypernatremia, and elevated serum creatinine. ROC analysis revealed good discriminative ability of the CAR, with an area under the curve of 0.841; a cutoff value of 0.44 yielded 78.1% sensitivity and 87.1% specificity.

Conclusions

Within this selected cohort of patients with NIHSS scores of 5-15, a higher early CAR was independently associated with unfavorable 30-day functional outcomes after spontaneous ICH. The identified cutoff should be considered cohort-specific and requires validation in unselected ICH populations across the full severity spectrum with 90-day follow-up.

## Introduction

Spontaneous intracerebral hemorrhage (ICH) represents a formidable global health challenge that contributes significantly to mortality and long-term disability worldwide [1-3]. Accounting for 10-30% of all strokes, ICH is particularly prevalent in Asian, Hispanic, and African American populations, with its incidence increasing in low- and middle-income countries (LMICs), such as Bangladesh, due to evolving demographics and suboptimal preventive healthcare measures [2]. The socioeconomic burden is substantial, exacerbated by limited access to neurocritical care and diagnostic delays in resource-constrained settings, which often lead to higher case fatality rates and prolonged postacute care dependency [4].

Accurate and early prognostication following acute ICH onset is critical for guiding patient management, stratifying risk for intensive interventions, and facilitating informed discussions with patients and their families [5]. However, conventional prognostic indicators, including baseline neurological assessments such as the Glasgow Coma Scale (GCS), as well as hematoma volume and location derived from noncontrast computed tomography (CT), frequently prove insufficient for precise risk prediction within the dynamic early phases of ICH [4,5]. Furthermore, advanced neuroimaging techniques, such as perfusion CT or MRI, which could offer more granular insights, are often unavailable in many LMIC clinical environments [4,6]. This deficiency underscores an urgent need for easily accessible, cost-effective, and rapidly obtainable blood-based biomarkers that can reliably reflect underlying pathophysiological processes and predict functional outcomes [5].

Systemic inflammation is increasingly recognized as a pivotal driver of secondary brain injury following ICH [7]. The extravasation of blood and subsequent breakdown products within the brain parenchyma triggers a robust innate immune response characterized by microglial activation and the infiltration of peripheral leukocytes [7]. This inflammatory cascade contributes to neuronal damage, perihematomal edema, and disruption of the blood‒brain barrier [7]. Previous studies have explored various inflammatory biomarkers, including C-reactive protein (CRP), the neutrophil-to-lymphocyte ratio (NLR), and cytokines, and demonstrated their associations with poor prognosis in hemorrhagic stroke patients, although with varying degrees of standardization and applicability in routine clinical practice [8].

CRP, an acute-phase reactant synthesized by the liver, rapidly increases in response to systemic inflammation, making it a sensitive indicator of inflammatory activity [9,10]. Conversely, albumin serves as a crucial marker of nutritional status and the systemic inflammatory response, with hypoalbuminemia often correlated with increased vascular permeability, compromised antioxidant defenses, and increased susceptibility to infection-related complications [9,11]. The C-reactive protein-to-albumin ratio (CAR) integrates these two complementary measures, providing a composite index that reflects both the intensity of systemic inflammation and the patient’s nutritional reserve [9,12]. This ratio has shown prognostic utility across diverse clinical contexts, including ischemic stroke, traumatic brain injury, sepsis, and critically ill pediatric patients, suggesting its broad relevance in conditions involving significant inflammatory and nutritional perturbations [9,10,12].

Despite the strong biological rationale and practical advantages of the CAR, prospective evidence evaluating its predictive value in spontaneous ICH, especially within South Asian populations or resource-limited healthcare settings, remains scarce [13]. This study, therefore, aims to address this critical knowledge gap by prospectively investigating the associations between the early CAR and short-term functional outcomes in patients with spontaneous ICH in a Bangladeshi cohort.

## Materials and methods

Study period and place

This prospective cohort study was conducted between February 2021 and January 2023 in the Department of Neurology at Dhaka Medical College & Hospital and the National Institute of Neurosciences & Hospital, Dhaka, Bangladesh.

Sample size calculation

A consecutive sampling technique was used to recruit participants. Eligible patients were enrolled consecutively after applying predefined inclusion and exclusion criteria and providing written informed consent. The sample size was calculated via the following formula:

\[ n = \frac{Z^{2}pq}{d^{2}} \]

Using a 95% confidence level (Z = 1.96), a precision (d) of 5%, and an expected proportion of unfavorable 30-day functional outcome (p) of 80.8% (q = 19.2%) based on a previous Bangladeshi cohort study by Tapu et al., which reported a good functional outcome in 19.2% of spontaneous ICH patients at one-month follow-up [14]. Therefore, the required minimum sample size was estimated to be approximately 239, rounded up to 240 patients. Accordingly, all eligible patients presenting consecutively during the study period were enrolled, yielding a total of 250 patients, which exceeded the calculated minimum requirement. After accounting for loss to follow-up (n = 30; 12%), 220 patients were included in the final analysis.

Patient selection

Eligible participants were adults aged ≥18 years with first-ever spontaneous ICH, presentation to the hospital within 72 hours of symptom onset and an admission National Institutes of Health Stroke Scale (NIHSS) score of 5-15. Consequently, the study population represented a restricted range of neurological severity and did not include patients with very mild (NIHSS <5) or severe neurological deficits (NIHSS >15). Patients were excluded if the hemorrhage was attributable to head trauma, hemorrhagic transformation of ischemic stroke or subarachnoid hemorrhage. Additional exclusion criteria included previous surgical or interventional treatment for the index event, current antiplatelet or anticoagulant therapy, bleeding diathesis, acute or chronic infection within four weeks preceding the index event, chronic kidney disease, congestive heart failure, chronic liver disease, respiratory failure, metabolic encephalopathy, malignancy, and pregnancy. A total of 250 eligible patients were initially enrolled. During follow-up, 30 patients did not have available 30-day outcome data: 18 could not be contacted despite repeated telephone attempts, and 12 were transferred to other institutions before completion of follow-up. Therefore, the final complete-case analysis included 220 patients with available 30-day functional outcome data.

Clinical variables and outcomes

Baseline data collected at admission included demographic characteristics, vascular risk factors (hypertension, diabetes mellitus, smoking and dyslipidemia), clinical features, GCS scores [15] and blood pressure measurements. Laboratory parameters included blood glucose, serum electrolytes, creatinine, CRP and serum albumin. Venous samples for CRP and albumin were obtained during the initial laboratory evaluation after hospital presentation, with all samples collected within 72 hours of symptom onset. The exact interval from hospital admission to venipuncture was not separately recorded; therefore, a mean or median sampling interval could not be calculated. The CAR was calculated by dividing CRP by serum albumin. Hemorrhage location, intracerebral hemorrhage volume, midline shift, and hydrocephalus were evaluated using CT imaging. Spontaneous ICH was defined as sudden intraparenchymal bleeding confirmed by CT in the absence of trauma or a structural lesion.

The GCS is commonly employed to evaluate neurological status and has been shown to predict mortality more effectively than conventional hemodynamic indicators such as systolic and diastolic blood pressure and heart rate. The total score is obtained by adding the eye-opening, verbal, and motor responses. While this composite measure provides a thorough appraisal of cerebral function, completing the assessment can be time intensive and may postpone immediate on-scene management. Stroke severity at presentation was evaluated via the NIHSS, which ranges from 0 to 42, with higher values reflecting greater neurological impairment [16].

Follow-up information and outcome evaluation

All patients received standard treatment according to American Heart Association guidelines [17] and were monitored throughout hospitalization. The primary endpoint was functional outcome at 30 days, which was assessed via the modified Rankin scale (mRS). The instrument categorizes patient status into seven stages: no symptoms (0), minor functional limitations (1), slight disability (2), moderate disability (3), moderately severe disability (4), severe disability (5), and death (6). The outcomes were categorized as favorable (mRS 0-3) or unfavorable (mRS 4-6) [18].

Statistical analysis

Data were analyzed using IBM SPSS Statistics for Windows, Version 26 (Released 2018; IBM Corp., Armonk, New York, United States). The distribution of continuous variables was assessed using the Shapiro-Wilk test and visual inspection of histograms. Normally distributed continuous variables are presented as mean ± standard deviation (SD), whereas nonnormally distributed variables are presented as median and interquartile range (IQR). Categorical variables are presented as number and percentage, n (%). Between-group comparisons were performed using the independent-samples t-test for normally distributed continuous variables, with the t statistic reported, and the Mann-Whitney U test for nonnormally distributed variables, with the U statistic reported. Categorical variables were compared using Pearson’s chi-square test, with the χ² statistic reported. Spearman’s rank correlation coefficient was used to assess the relationship between the CAR and 30-day mRS scores. Multivariable binary logistic regression was performed to identify independent predictors of unfavorable outcomes, and results are presented as adjusted odds ratios (AORs), 95% confidence intervals (CIs), and p-values. Receiver operating characteristic curve analysis was performed to evaluate the discriminatory performance of the CAR. The optimal cutoff was selected using the Youden index. All tests were two-tailed, and p<0.05 was considered statistically significant. Values below 0.001 were reported as p<0.001. The primary analysis followed a complete-case approach and included participants with available 30-day mRS data; missing outcome data were not imputed.

Ethical consideration

The study was conducted in accordance with the ethical principles outlined in the Declaration of Helsinki. Ethical approval was obtained from the Ethical Review Committee of Dhaka Medical College (Approval No.: ERC-DMC/ECC/2021/10). Before enrollment, written informed consent was obtained from all participants.

## Results

Baseline sociodemographic and vascular characteristics

Among the analyzed patients, 90 (40.9%) had favorable outcomes, and 130 (59.1%) had unfavorable outcomes. Patients with unfavorable outcomes were significantly older than those with favorable outcomes (62.1 ± 14.2 vs. 52.7 ± 12.2 years; p < 0.001). Sex, residence, hypertension, diabetes mellitus, and dyslipidemia did not differ significantly between the groups. Smoking was more common among patients with unfavorable outcomes than among those with favorable outcomes (70/130 (53.8%) vs. 36/90 (40.0%); p=0.043) (Table 1).

**Table 1:** Baseline sociodemographic and vascular risk factors by 30-day outcome (n = 220) Data are presented as mean ± standard deviation (SD) or n (%). Age was compared using the independent-samples t-test, whereas categorical variables were compared using Pearson’s chi-square test. A two-tailed p<0.05 was considered statistically significant; values below 0.001 are reported as p<0.001. SD: standard deviation.

Variable	Category	Favorable (n=90)	Unfavorable (n=130)	t/χ²	p-value
Age	Mean ± SD	52.7±12.2	62.1±14.2	-5.11	<0.001
Sex	Male	45 (50.0)	69 (53.1)	0.202	0.653
	Female	45 (50.0)	61 (46.9)		
Residence	Urban	32 (35.6)	43 (33.1)	0.145	0.730
	Rural	58 (64.4)	87 (66.9)		
Hypertension	Yes	74 (82.2)	113 (86.7)	0.922	0.337
	No	16 (17.8)	17 (13.3)		
Smoking status	Yes	36 (40.0)	70 (53.8)	4.084	0.043
	No	54 (60.0)	60 (46.2)		
Diabetes	Yes	15 (16.7)	15 (11.5)	1.188	0.276
	No	75 (83.3)	115 (88.5)		
Dyslipidemia	Yes	11 (12.2)	14 (10.8)	0.111	0.738
	No	79 (87.8)	116 (89.2)		

Neurological and radiological characteristics at admission

Patients with unfavorable outcomes had lower admission GCS scores (9.8 ± 0.9 vs. 10.1 ± 0.9; p = 0.016) and higher NIHSS scores (7.6 ± 2.0 vs. 6.8 ± 1.8; p = 0.004). Systolic blood pressure at admission was also higher in the unfavorable group (168.1 ± 24.1 vs. 159.2 ± 30.8 mmHg; p = 0.018). In terms of imaging findings, supratentorial hematoma location was more common among patients with unfavorable outcomes than among those with favorable outcomes (119/130 (91.5%) vs. 73/90 (81.1%); χ²=5.206, p=0.023). Left-sided lesions (74/130 (56.9%) vs. 25/90 (27.8%)), intraventricular hemorrhage (83/130 (63.8%) vs. 40/90 (44.4%)) and hydrocephalus (58/130 (44.6%) vs. 25/90 (27.8%)) were also more common in the unfavorable-outcome group (Table 2).

**Table 2:** Admission neurological and radiological characteristics by 30-day outcomes (n = 220) Data are presented as mean ± standard deviation (SD) or n (%). Glasgow Coma Scale, National Institutes of Health Stroke Scale and systolic blood pressure were compared using the independent-samples t-test. A two-tailed p<0.05 was considered statistically significant; values below 0.001 are reported as p<0.001. GCS: Glasgow Coma Scale; NIHSS: National Institutes of Health Stroke Scale; SBP: systolic blood pressure; SD: standard deviation.

Characteristic	Favorable	Unfavorable	t/χ²	p-value
Neurological parameters				
GCS (mean ± SD)	10.1±0.9	9.8±0.9	2.431	0.016
NIHSS (mean ± SD)	6.8±1.8	7.6±2.0	-3.042	0.004
SBP (mmHg) (mean ± SD)	159.2±30.8	168.1±24.1	-2.401	0.018
Radiological characteristics				
Supratentorial location, n (%)	73 (81.1)	119 (91.5)	5.206	0.023
Left-sided lesion, n (%)	25 (27.8)	74 (56.9)	18.253	<0.001
Intraventricular hemorrhage, n (%)	40 (44.4)	83 (63.8)	8.121	0.004
Hydrocephalus, n (%)	25 (27.8)	58 (44.6)	6.418	0.011

Laboratory biomarkers

From samples obtained during the initial laboratory evaluation after hospital presentation and within 72 hours of stroke onset, serum creatinine levels were modestly higher among patients with unfavorable outcomes (median 0.9 (0.7-1.3) vs. 0.8 (0.7-1.2) mg/dL; p = 0.035). Median CRP concentrations were markedly elevated in the unfavorable group (18.0 (7.0-38.5) vs. 7.5 (4.4-10.7) mg/L; p < 0.001), whereas serum albumin levels did not differ significantly between the groups (p = 0.297). The CAR was significantly higher in patients with unfavorable outcomes (0.44 [0.10-1.00] vs. 0.20 [0.10-0.30]; p < 0.001) (Table 3).

**Table 3:** Laboratory biomarkers obtained within 72 hours of stroke onset by 30-day outcomes (n = 220) Data are presented as median and interquartile range (IQR). Groups were compared using the Mann-Whitney U test. A two-tailed p<0.05 was considered statistically significant; values below 0.001 are reported as p<0.001. CRP: C-reactive protein; CAR: C-reactive protein-to-albumin ratio; IQR: interquartile range.

Biomarker	Favorable	Unfavorable	p-value
	median (IQR)	median (IQR)	
Creatinine	0.8 (0.7–1.2)	0.9 (0.7–1.3)	0.035
CRP	7.5 (4.4–10.7)	18.0 (7.0–38.5)	<0.001
Albumin	37.0 (35–40)	39.2 (35–42)	0.297
CAR	0.20 (0.10–0.30)	0.44 (0.10–1.00)	<0.001

Correlation between the CAR and functional outcome

**Figure 1:**
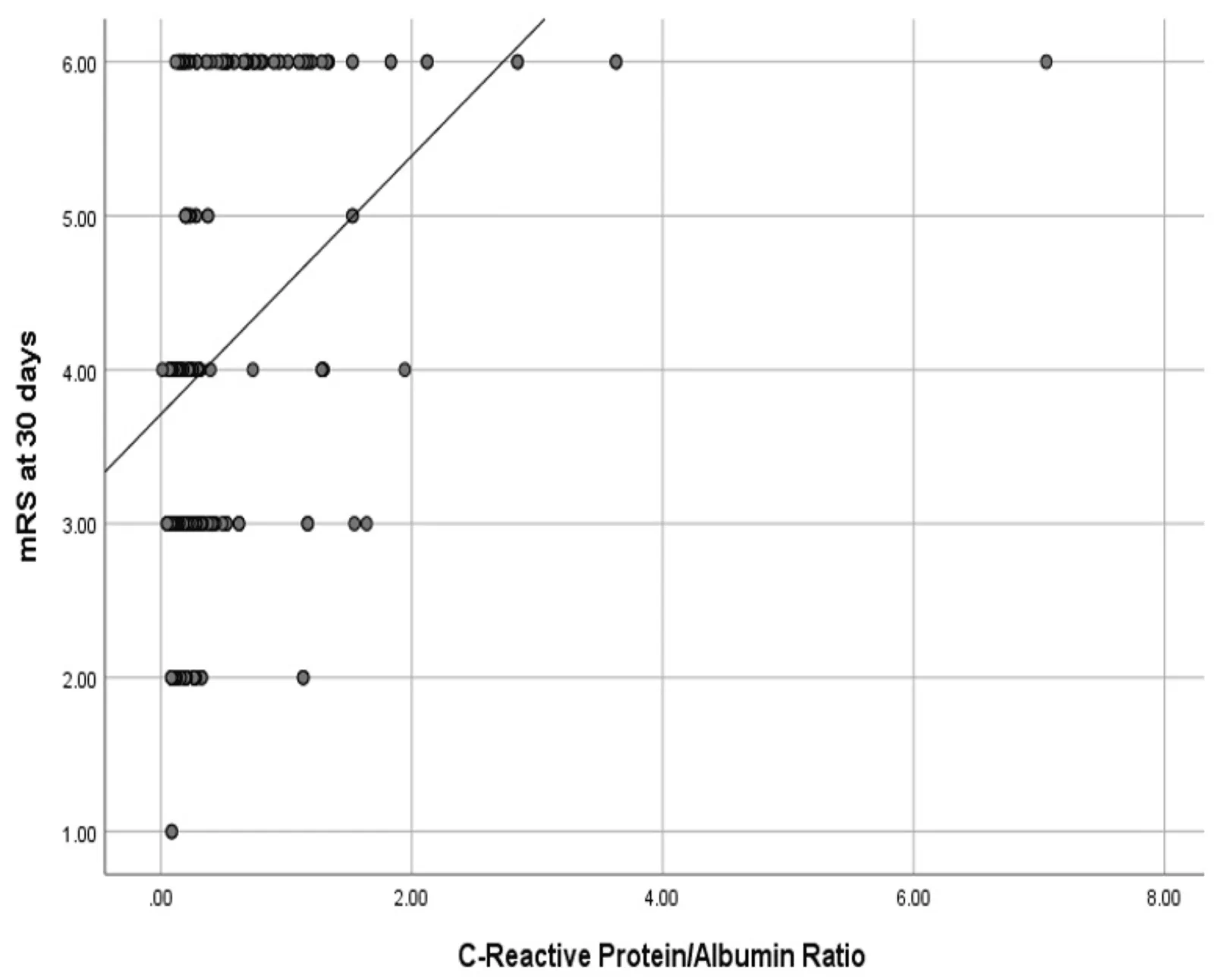
Correlation between the mRS score and CAR (n=220) mRS: Modified Rankin scale; CAR: C-reactive protein-to-albumin ratio

Spearman correlation analysis revealed a moderate correlation between the CAR score and the 30-day mRS score (rs = 0.513, p < 0.001) (Figure 1).

Multivariable predictors of unfavorable outcomes

In the multivariable logistic regression analysis, increasing age was independently associated with unfavorable outcomes (adjusted OR 1.07 per year; 95% CI 1.04-1.11; p < 0.001). Other independent predictors included hemiparesis (OR 7.05; 95% CI 1.65-30.11; p = 0.008), aspiration pneumonia (OR 4.63; 95% CI 1.64-13.10; p = 0.004), hypernatremia (OR 14.34; 95% CI 2.26-91.16; p = 0.005), elevated serum creatinine (OR 2.88; 95% CI 1.15-7.19; p = 0.024), and CAR (OR 4.51; 95% CI 1.63-12.50; p = 0.004). Left-sided lesion location was significantly associated with unfavorable outcomes (OR 1.18; 95% CI 1.12-2.44; p < 0.001) (Table 4).

**Table 4:** Multivariable logistic regression for predictors of unfavorable 30-day outcomes Results are presented as adjusted odds ratios (AORs) with 95% confidence intervals (CIs). Multivariable logistic regression analysis was done. A two-tailed p<0.05 was considered statistically significant; values below 0.001 are reported as p<0.001. AOR: adjusted odds ratio; CI: confidence interval; CAR: C-reactive protein-to-albumin ratio.

Predictor	Adjusted OR	95% CI	p-value
Age	1.07	1.04–1.11	<0.001
Hemiparesis	7.05	1.65–30.11	0.008
Left-sided lesion	1.18	1.12–2.44	<0.001
Aspiration pneumonia	4.63	1.64–13.10	0.004
Hypernatremia	14.34	2.26–91.16	0.005
Creatinine	2.88	1.15–7.19	0.024
CAR	4.51	1.63–12.50	0.004

Diagnostic performance of the CAR

Receiver operating characteristic curve analysis demonstrated good discriminative ability of the CAR for predicting unfavorable 30-day outcomes, with an area under the curve of 0.841 (95% CI 0.785-0.898). The optimal cutoff value of 0.44 yielded a sensitivity of 78.1% and a specificity of 87.1%, with a Youden index of 0.65 (Table 5).

**Table 5:** Diagnostic performance of the CAR for predicting unfavorable outcomes Diagnostic performance is presented as the cutoff, sensitivity, specificity, Youden index and area under the curve with 95% confidence interval. A two-tailed p<0.05 was considered statistically significant. AUC: area under the curve; CI: confidence interval; CAR: C-reactive protein-to-albumin ratio.

Cutoff	Sensitivity (%)	Specificity (%)	Youden index	AUC (95% CI)
0.44	78.1	87.1	0.65	0.841 (0.785–0.898)

## Discussion

In the present prospective cohort, the CAR was independently associated with unfavorable 30-day functional outcomes following spontaneous ICH among patients with admission NIHSS scores of 5-15 in a South Asian LMIC setting. This finding supports the potential prognostic relevance of CAR as an integrated marker of systemic inflammation and nutritional status, although it does not establish clinical utility across the full spectrum of ICH severity [13,19]. A substantial proportion of patients experienced unfavorable outcomes, consistent with the considerable burden of stroke-related morbidity and mortality in LMICs [5].

Another important observation was that CAR, specifically, acts as a robust indicator of the interplay between inflammation and nutritional compromise. Previous studies reported the same findings [11,20]. The CRP component of the CAR reflects the magnitude of innate immune activation, which is driven primarily by interleukin-6-mediated hepatic CRP synthesis during the acute phase of the response [21]. Conversely, albumin levels can decrease due to capillary leakage, reduced hepatic synthesis, or catabolic stress following brain injury, which is a common phenomenon in critically ill patients [11]. Unlike isolated CRP or albumin, the CAR captures the dynamic imbalance between the proinflammatory drive and host resilience, offering superior prognostic discrimination over conventional inflammatory indices such as the NLR or platelet-to-lymphocyte ratio in hemorrhagic stroke cohorts [22,23]. Although the level of serum ALB alone did not differ significantly between the outcome groups in our cohort, its interaction with CRP within the CAR composite may better reflect the inflammatory-nutritional imbalance driving secondary brain injury. Studies in East Asian spontaneous ICH populations have similarly reported that the CAR is a stronger predictor of mortality than CRP alone, and data from European neuro-intensive care units confirm its incremental value beyond clinical scores in early risk stratification [12,24]. Furthermore, the CAR has been identified as a valuable prognostic marker in diverse conditions, including peripheral artery disease and COVID-19, highlighting its broad applicability in critical care settings [20].

The results from our study further demonstrated that patients with unfavorable outcomes presented lower admission GCS scores and higher NIHSS scores. These findings are consistent with previous studies identifying admission neurological severity as an important prognostic indicator in spontaneous ICH [25,26]. Additionally, imaging findings revealed that supratentorial hematoma locations were more common in patients with unfavorable outcomes, as were left-sided lesions, intraventricular hemorrhage, and hydrocephalus. These observations echo findings from multicenter registries in various global regions, where such neurological and radiological indicators are well-established predictors of poor prognosis in ICH patients [25]. For example, hematoma volume, which often correlates with lesion location and extent, is a critical determinant of outcome [26]. The presence of intraventricular hemorrhage is also a known risk factor for worse outcomes because of its association with hydrocephalus and increased intracranial pressure [27].

This study also revealed that aspiration pneumonia, hypernatremia, and elevated serum creatinine were strongly associated with unfavorable outcomes. These complications mirror patterns observed in large ICU-based ICH cohorts globally, where pulmonary and renal dysfunction significantly mediate secondary injury and prolonged disability [28]. Aspiration pneumonia, for example, is a frequent complication of stroke that can significantly worsen patient prognosis, a fact supported by research from Asian stroke units [8]. The development of acute kidney injury, as indicated by elevated serum creatinine, is also known to contribute to increased mortality in spontaneous ICH patients [28]. Because CRP may also increase in response to pulmonary infection, an elevated CAR may partly reflect early or subclinical aspiration rather than ICH-related neuroinflammation alone. Moreover, 30-day mRS may be influenced by acute medical complications and may not represent stable neurological disability. Therefore, assessment at 90 days is required to confirm the longer-term prognostic value of the CAR.

Advancing age was independently associated with an unfavorable outcome in this cohort, which is consistent with global epidemiological trends [29]. Furthermore, smoking was more common among patients with unfavorable outcomes, extending prior evidence from South Asian populations regarding heightened cerebrovascular vulnerability among tobacco users [30]. The high prevalence of smoking and smokeless tobacco use in this region may amplify endothelial dysfunction and coagulopathy, exacerbating stroke severity and outcomes [30]. While hypertension and diabetes mellitus are well-known risk factors for stroke, their direct differential impact on functional outcomes in this specific cohort did not reach statistical significance between the favorable and unfavorable groups, suggesting that other factors may play a more immediate role in acute outcome prediction in this setting [30]. The CAR demonstrated good discriminative ability for unfavorable 30-day outcomes within the present cohort, with an area under the curve of 0.841. However, enrollment was restricted to patients with admission NIHSS scores of 5-15, which may have influenced the AUC, sensitivity, specificity, and optimal cutoff. Therefore, the cutoff of 0.44 should be considered cohort-specific and should not be extrapolated to an unselected ICH population. External validation across the full spectrum of ICH severity with 90-day follow-up is required before routine clinical application [12,18].

This prospective two-center study evaluated a low-cost, routinely available biomarker in a South Asian LMIC setting and incorporated clinical, laboratory, and radiological variables. Nevertheless, the observational design precluded causal inference. Restriction to patients with NIHSS scores of 5-15 excluded very mild and severe ICH, limiting generalizability and the applicability of the identified cutoff. In addition, 30 of 250 participants (12.0%) were lost to follow-up. A formal baseline comparison with these patients could not be performed because their individual-level data were unavailable at revision, and potential attrition bias cannot be excluded. Exact admission-to-venipuncture intervals and serial biomarker measurements were also unavailable. Finally, 30-day mRS may have been influenced by acute complications, particularly aspiration pneumonia, rather than stable stroke-related disability.

## Conclusions

In patients with spontaneous ICH, a higher early CAR ratio was independently associated with unfavorable short-term functional outcomes. The ratio may serve as a simple, low-cost adjunct for early risk stratification, although validation in broader multicenter populations is required before its routine clinical application.
